# Serum Copper and Haematological Values of Sheep of Different Physiological Stages in the Dry and Wet Seasons of Central Trinidad

**DOI:** 10.1155/2014/972074

**Published:** 2014-05-08

**Authors:** A. Mohammed, M. Campbell, F. G. Youssef

**Affiliations:** ^1^Eastern Caribbean Institute of Agriculture and Forestry, Faculty of Biosciences, Agriculture and Food Technology, University of Trinidad and Tobago, Caroni North Bank Road, Centeno Arima, Trinidad and Tobago; ^2^School of Veterinary Medicine, Faculty of Medical Sciences, The University of the West Indies (UWI), Mount Hope, Trinidad and Tobago; ^3^Department of Food Production, UWI, Saint Augustine, Trinidad and Tobago

## Abstract

A study was conducted to determine serum copper (Cu) concentrations and hematological values of 174 sheep from four medium sized farms, pertaining to physiological stage, in the late dry and late wet seasons of Central Trinidad. Serum Cu was significantly lower in the dry than in the wet season (*P* < 0.001) with a corresponding high percentage of samples with low Cu levels in the former. 31% of dry nonpregnant sheep exhibited a nonregenerative anaemia. Also, hemoglobin and packed cell volume values varied (*P* < 0.001) including lymphocyte (*P* < 0.01) counts, among growing animals compared with other physiological stages. Significant variations also occurred among neutrophil (*P* < 0.05) and eosinophil (*P* < 0.05) values in sheep. Highest haemoglobin and packed cell volume, white blood cell counts, and lymphocyte values in growing sheep compared with other stages were probably age related.

## 1. Introduction

Grazing ruminants depend largely on native and improved forages of pastures to supply their nutrient requirements for growth and production. However, tropical pastures are frequently deficient in energy, protein, and minerals with the culminating effect of low animal productivity. Both phosphorus (P) and copper (Cu) are the major minerals limiting production of grazing animals in African, Asian, and Latin American countries [1]. Copper deficiencies in ruminants has been associated with depressed growth, bone disorders, severe diarrhea, infertility, cardiac failure, enzootic ataxia, hair depigmentation, susceptibility to bacterial infections, and anaemia. These disorders are closely related to the functional roles of Cu-containing enzymes in cellular respiration, membrane stability, immune function, and the formation of the red blood cells (erythropoiesis).

Cu is required for the function of over 30 proteins including superoxide dismutase, ceruloplasmin, lysyl oxidase, cytochrome c oxidase, tyrosinase, dopamine-*β*-hydroxylase, and hephaestin [2]. Copper functions as an electron transfer intermediate in redox reactions and is, therefore, an essential cofactor in oxidative and reductive enzymes. Hephaestin, cytochrome C oxidase (EC 1.9.3.1), and ceruloplasmin (EC 1.16.3.1) are required for iron absorption and transport and are, therefore, closely required in erythropoiesis. Hephaestin is involved in the release of iron from intestinal enterocytes and into the blood [2]. Cytochrome c oxidase is involved in the reduction of ferric iron to the ferrous form for use in haem synthesis. Ceruloplasmin (Cu oxidase) facilitates mobilization of iron from the liver and tissues other than enterocytes [3] as well as binding of iron to the iron transport protein transferring [2]. Cu deficiency results in decreased production of these Cu-containing mitochondrial proteins and, in turn, affects functional iron deficiency. Affected animals generally have microcytic hypochromic anaemia [3]. It has also been found that reduced Cu dependent cytochrome C oxidase activity in liver hepatocytes and bone marrow erythroid cells contribute to impaired haem synthesis which may explain the microcytic, hypochromic anaemia sometimes observed in Cu deficiency [4]. Note, however, that the feeding of semipurified Cu-deficient diets (<1 mg Cu/kgDM) to lambs and calves can result in a marked or marginal decline in haemoglobin and packed cell volume levels [5, 6]. A copper deficient diet is synonymous with a serum Cu critical level of less than 0.5 mg/L [1].

Red blood cell (RBC) counts and packed cell volume (PCV) are influenced not only by nutritional compromise conditions but also by season [7, 8], age [9], parasitic infection [10], and physiological stage [3]. Adewuyi and Adu [7] found low PCV and haemoglobin (Hb) values in certain West African sheep breeds, at the end of the rainy season. Note, however, that low PCV values in sheep and goats in the tropics during the rainy season are sometimes associated with increased parasitic infection at this time [11, 12].

Regarding age and physiological stage, one study in sheep and another in goats demonstrated higher Hb and PCV values in lambs and kids at 6–12 months of age compared with values in pregnant sheep and goats [13, 14]. However, Hb values and PCV values, sequentially monitored in goats and sheep during pregnancy and onwards, generally reflect a rise in these values in late gestation followed by a decline in the periparturient period and a further decline in early lactation [15–17]. Contrastingly, in West African Dwarf sheep, Obidike et al. [18] did not find significant change in these values in the periparturient period except a rise at two weeks after parturition. The decline in Hb and PCV values in late gestation is attributed to an increase in plasma volume [3]. The decline in early to midlactation in ruminants is related to milk production [19, 20].

The white blood cell (WBC) and differential leukocyte counts in sheep and goats are also subject to variations due to age, physiologic stage, and parasitic infection. Thus, higher WBC and lymphocyte counts are found in kids less than 12 months of age compared with values in adult goats [14, 21]. Additionally, total WBC may be elevated in late pregnancy in sheep [22] and goats [23] because of an ACTH-related hormonal stress reaction. Eosinophilia and sometimes basophilia in growing and adult animals may be indicative of an allergic response to recent parasitic infection [14].

In a farm investigative study of swayback prone farms of Central Trinidad, Mohammed [24] found that affected kids and lambs had a nonregenerative anaemia related to Cu deficiency. However, it is not known whether a relationship exists between hematological values and blood serum Cu levels on goat and sheep farms of Central Trinidad, from which have been no reported cases of swayback due to Cu deficiency. The purpose of this research was to study the effects of season and physiological stage on serum Cu levels and on certain haematological and plasma parameters of sheep. This study can provide useful information on whether hematological values of apparently normal ruminants are affected by season and physiological stage in relation to dietary Cu sufficiency.

## 2. Materials and Methods

### 2.1. Ethics Approval

The research protocols for this study were approved by the Veterinary Ethics committee of the Faculty of Medical Sciences, The University of the West Indies.

### 2.2. Farms and Management

**Figure 1:**
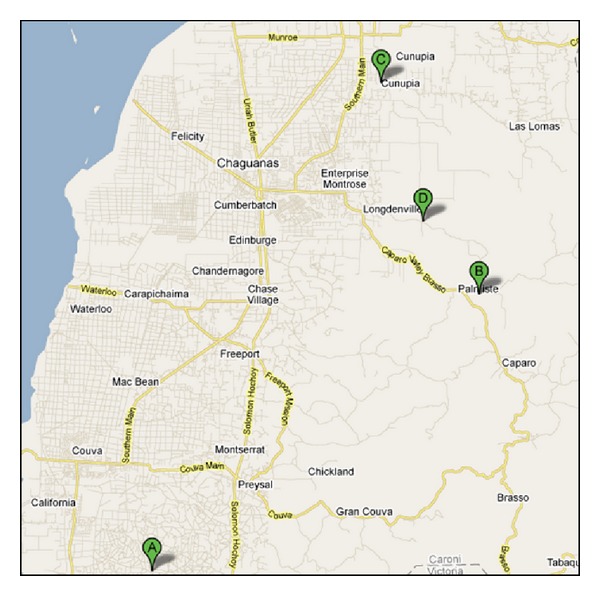
Blood sampling at farm locations of Central Trinidad. A: Couva farm; B: Palmiste farm; C: Cunupia farm; D: Sugarcane Feeds Centre, Longdenville.

This study was carried out in the late dry (April-May) and late wet (November-December) seasons on four local farms of Central Trinidad. These included the state owned Sugarcane Feeds Centre of Longdenville and the Mon Jaloux Livestock Farm of Chin Chin, respectively, and two private farms from the Cunupia and Palmiste farms of the Central region (Figure 1). The first two farms were situated on a fine sandy loamy soil, while the latter two were situated on fine sandy clay, a sandy clay loam, and fine sandy soil types. Maximum monthly rainfall in the late dry and late wet seasons from the five farms ranged from 25 to 53 mm and from 42 to 191 mm, respectively. Correspondingly, minimum and maximum temperatures ranged from 22 to 34°C and from 20 to 32°C, respectively. Sheep were reared intensively at the Sugarcane Feeds Centre and Mon Jaloux Livestock farms and semi-intensively at the Cunupia and Palmiste farms. Sheep were mainly of Barbados Black Belly and to a lesser extent of West African hair type origins.

Except for the Mon Jaloux Livestock Farm, sheep at other farms were either zero grazed or allowed grazing mainly on forages bamboo grass (*Paspalum fasciculatum*), elephant grass (*Pennisetum purpureum*), and tapia grass (*Sporobolus indicus*). Sheep at the Mon Jaloux livestock Farm, the Cunupia, and Palmiste were also fed a urea-molasses, bagasse mixture.

Sheep on all farms were not managed along a set pattern of deworming procedures. However, sheep at all farms were occasionally dewormed at weaning. Animals were either dewormed orally using fenbendazole, at a dose rate of 5 mg/kg (Anglian nutrition Products Co., Ipswich, UK), or by subcutaneous injection with ivermectin (1% m/v) at a rate of 200 *μ*g/kg (ECO Animal Health Southern Africa Ltd., Northmead, Gouteng).

### 2.3. Sample Collection

Blood samples were collected from 174 sheep in the late dry (*n* = 86) and late wet seasons (*n* = 88) of Central Trinidad. Sheep blood samples were collected from the Cunupia, Palmiste, the Sugarcane Feeds Centre, and Mon Jaloux Livestock farms. Blood samples were collected in the two seasons from different growing (6–12 mth), dry pregnant (last trimester), early lactating (up to 8 wk), and dry nonpregnant sheep (1–3 yrs). Blood samples were taken from different apparently healthy animals at the same farm at the end of the dry and wet seasons, respectively, according to the availability of animals by physiological stage. Approximately 25% of the total sheep at the Mon Jaloux Livestock Farm and 50–60% of sheep at other farms were surveyed via blood sampling.

Blood for complete blood counts (CBC) was drawn by venipuncture using 16–18 mm gauge needles and collected in 5 mL bottles containing approximately 0.5 mg/mL K_2_EDTA (potassium ethylene diamine tetra acetic acid) as an anticoagulant. The anticoagulated blood was used for manual complete blood counts (CBCs) and plasma protein determination. Five milliliters of blood was also collected from each animal in acid-washed demineralised tubes and allowed to clot. Serum was then collected via centrifugation within 4 hours of blood collection and stored at −20°C.

### 2.4. Hematology

The PCV (L/L) was determined by the microhematocrit method from Schalm haematology [3]. A Coulter hemoglobinometer method was used to determine Hb concentration (g/L). Total white blood cell (WBCs) counts were performed manually using a hemocytometer. Total plasma protein values (g/L) were obtained using a Goldberg refractometer. Plasma fibrinogen levels (g/L) were determined by Millar's heat precipitation method [3]. The differential leukocyte count (10^9^/L) was performed on a Wright's-Giemsa stained blood smear according to Thrall et al. [25]. Reticulocyte percentages were calculated for animals with low haemoglobin (<8.0 g/L) and PCV (<22%) values according to Thrall et al. [25]. The stained blood smears from all animals were also examined for morphological abnormalities. Faecal egg counts in relation to RBC value were not done as part of this study.

### 2.5. Copper Analysis

Serum Cu levels were determined according to Fick et al. [26] using a Pye Unicam 2900 Atomic Absorption Spectrophotometer equipped with a PU9090 data graphics system.

### 2.6. Statistical Analysis

Hematological parameter means were tested between seasons and among physiological stages in goats by analysis of variance using the GenStat Release 13.3, Copyright 2010 VSN International Ltd. Significantly different means were compared using the protected Fishers LSD (*P* < 0.05) test.

## 3. Results

### 3.1. Serum Cu and Hematological Values of Sheep in the Dry and Wet Seasons

Serum Cu levels varied with season in sheep (*P* < 0.001) but not physiological stage (*P* > 0.05). Serum Cu was significantly lower in the dry than in the wet season (*P* < 0.001) with a higher percentage of serum Cu below critical level (BCL) (<0.5 mg/L) in the end of the dry (60%) than at the end of the wet (30%) season. In sheep, hemoglobin and packed cell volume values were not significantly different (*P* > 0.05) between seasons (Table 1). However, sheep had somewhat similar percentages of low haemoglobin and packed cell volume values in the dry (15%) and wet (20%) seasons. Low haemoglobin and packed cell volume values in sheep ranged from 45 to 75 g/L and from 0.13 to 0.21 L/L, respectively, in both seasons. Animals with low haemoglobin and packed cell volume values in the wet and dry seasons also displayed low reticulocyte counts (<1%).

### 3.2. White Blood Cell and Differential Counts

Sheep had higher total white blood cell (*P* < 0.001), lymphocyte (*P* < 0.05), neutrophil (*P* < 0.05), and monocyte (*P* < 0.001) counts in the dry than in the wet season (Table 1). Sheep also had a higher percentage (49% and 26%) of mild leukocytosis (white blood cell count >12 × 10^9^/L) in the dry than in the wet season.

### 3.3. Serum Cu and Hematological Values of Sheep among Physiological Stages

Serum Cu did not vary among physiological stages in sheep (*P* > 0.05). However, high percentages of serum Cu below critical level (<0.5 mg/L) were found at all physiological stages in sheep (40–60%) [1, 27]. Hemoglobin and packed cell volume values varied (*P* < 0.001) among physiological stages in sheep (Table 1). Growing lambs had higher (*P* < 0.001) haemoglobin and packed cell volume values than those dry nonpregnant sheep. However, haemoglobin and packed cell volume values were different neither between growing and lactating animals nor between dry pregnant and dry nonpregnant sheep (LSD *P* > 0.05). Dry nonpregnant sheep had the highest percentage (31%) of nonregenerative anaemia evident by low haemoglobin (<80 g/L), packed cell volume (<0.22 L/L) values, and a low reticulocyte count (<1%), when compared to the anaemia found in growing (5%), pregnant (20%), and lactating animals (15%). However, 9% of all sheep displayed mild acanthocytosis (+1 to +2) as the only evidence of red blood cell abnormality (poikilocytosis). The presence of acanthocytes is evidenced as abnormal projections on the red blood cells.

### 3.4. White Blood Cell and Plasma Protein Components among Physiological Stages

In sheep, variations occurred in the differential leucocyte counts for neutrophil (*P* < 0.05), lymphocyte (*P* < 0.01), and eosinophil (*P* < 0.05) values among the different physiological stages. A physiological x season interaction was observed for basophil (*P* < 0.01) and lymphocyte (*P* < 0.05) values (Table 2). These interactions due to higher values were found in growing lambs in the dry season compared to lower values in all other physiological stages (LSD *P* < 0.05) pertaining to season. Total WBC and lymphocyte counts were highest in growing lambs compared with other physiological stages (LSD *P* < 0.05).

44% of growing lambs had a mild leukocytosis (>12.0 × 10^9^/L), and 15% had a lymphocytosis (>9.0 × 10^9^/L). A mild leukocytosis was also present in pregnant (35%) and lactating sheep (37%). Lowest neutrophil but highest eosinophil values were found in dry nonpregnant sheep, 26% of which showed evidence of an eosinophilia (>1.0 × 10^9^/L) [27].

Lymphocyte: neutrophil ratios were highest in growing lambs (1.5) and declined in the pregnant (1.2) and lactating (1.0) stages but rose again in dry nonpregnant sheep (1.4). Highest plasma protein (*P* < 0.05) levels were found in dry nonpregnant sheep compared to those of other physiological stages.

## 4. Discussion

In this study, a high percentage of sheep sera were found to be below critical level indicative of a dietary insufficiency [1, 27]. Cu insufficiency in sheep animals could have affected growth, performance, and their proneness to disease inclusive of the nonregenerative anaemia manifested in both seasons. In a previous study of an outbreak of swayback in Central Trinidad, low serum Cu levels were associated with a terminal nonregenerative anaemia in affected kids and lambs [24]. A higher percentage of deficient serum Cu was also found in sheep at the end of the dry compared with the end of the wet season and was indicative of insufficient dietary Cu intakes. Only a few sheep exhibited normal serum Cu levels.

Serum Cu concentrations, within normal reference ranges, are present in plasma mainly in the form of the enzyme ceruloplasmin (EC1.16.3.1.), copper oxidase. Reduced ceruloplasmin activity, required for the binding of Iron III transferrin for erythropoiesis, could have been limited due to Cu deficiency [2]. The copper containing enzyme activity of cytochrome C oxidase (EC 1.9.3.1) may have also been reduced in liver hepatocytes and bone marrow erythroid cells resulting in impaired haem synthesis for erythropoiesis [4]. Additionally ceruloplasmin functional role as a transport protein used in the hepatic synthesis of cytochrome oxidase (EC 1.9.3.1) could have also been compromised [28]. Although the anaemia of copper deficiency is usually a long term consequence, in this study, there was a possible association, though not significant, between deficient Cu levels and the anaemia manifesting in both seasons and at various physiological stages in sheep [5]. Notwithstanding, an eosinophilia in dry nonpregnant sheep can be indicative of an allergic response possibly due to recent parasitism [3, 29].

Although haemoglobin and packed cell volume values were not different between late pregnant and early lactating sheep, values in sequentially monitored animals generally reflect mostly a rise in these values in late gestation, declining in the periparturient period and declining further in early lactation [15–17]. Note that El-Sherif and Assad [16] reported a rise in these values in late pregnancy in Egyptian Barki ewes compared with values in dry ewes. However, in this study, these values were not different between dry late pregnant and dry nonpregnant sheep. Animals in sequential studies are usually managed under experimental conditions such as similar diets and adequate anthelmintic control which was not controlled in this study. The anemia found in pregnant and lactating sheep is also probably related to an increase in plasma volume in late gestation and to milk yield changes in early lactation [3, 14, 20]. Also, mild acanthocytosis observed in a low percentage of sheep maybe an occasional finding characteristic of sheep or may be dietary related [25].

The highest haemoglobin and packed cell volume, white blood cell counts, and lymphocyte values found in growing sheep compared to values in other physiological stages are probably age related [13, 14, 21, 30]. The decline of neutrophil values in dry nonpregnant sheep was probably due to a lesser imposition of stress in these animals. The mild leukocytosis found in sheep is probably stress related and is also reported in similar studies in the tropics [18, 20, 23]. Most sheep of all physiological ages had plasma fibrinogen levels within expected ranges [3].

## 5. Conclusions

The purpose of this research was to study the effects of season and physiological stage on serum Cu levels and on certain red and white blood cell and plasma parameters of apparently normal sheep of Central Trinidad. There was a higher percentage of deficient serum Cu found in sheep at the end of the dry compared with the end of the wet season which was also indicative of insufficient dietary Cu intakes. Hence, there was a possible association between deficient Cu levels and the anaemia manifesting in both seasons in sheep, respectively. The anaemia manifested mostly at adult physiological stages in both species. Dietary Cu insufficiency in affected animals could have affected growth, performance, and their proneness to disease inclusive of the anaemia observed in this study. An eosinophilia in dry nonpregnant sheep also suggested parasitic infection contributing to the observed anaemia in these animals. A recommendation is made for copper supplementation and adequate parasitic control measures for sheep of Central Trinidad.

## Figures and Tables

**Table 1:** Hematological values and serum copper levels of sheep of different physiological stages in the dry and wet seasons of Central Trinidad.

Blood	Season				Physiological stage										
Parameters	Dry		Wet		Growing		Dry pregnant		Lactating		Dry nonpregnant		^ x^Significance		
*N*	86		88		41		45		46		42				

Parameter	Mean	SE	Mean	SE	Mean	SE	Mean	SE	Mean	SE	Mean	SE	Season	Phys. stage	Phys. × sea.

Red blood cells															
Hb (g/L)	94.1	1.63	93.4	1.61	100.8^a^	2.35	90.2^bc^	2.25	96.0^ab^	2.22	88.2^c^	2.33	NS	∗∗∗	NS
PCV (L/L)	0.28	0.01	0.28	0.01	0.30^a^	0.01	0.27^bc^	0.01	0.28^ab^	0.01	0.26^c^	0.01	NS	∗∗∗	NS
MCHC (g/L)	335	0.90	337	0.90	335	1.30	337	1.20	337	1.20	336	1.30	NS	NS	NS
Serum Cu (mg/L)	0.42^c^	0.02	0.57^d^	0.02	0.50	0.03	0.52	0.023	0.52	0.03	0.44	0.03	∗∗∗	NS	NS
White blood cells (×10^9^/L)															
WBC	11.7^c^	0.39	9.9^d^	0.39	11.9	0.57	10.6	0.54	10.9	0.54	9.7	0.56	∗∗∗	NS	NS
Neutrophil	4.77^c^	0.23	4.00^d^	0.28	4.52^a^	0.33	4.42^ab^	0.32	4.92^a^	0.31	3.61^b^	0.33	∗	∗	NS
Lymphocyte	5.81^c^	0.25	5.00^d^	0.25	6.60^a^	0.37	5.18^b^	0.35	4.89^b^	0.35	5.01^b^	0.36	∗	∗∗	∗
Eosinophil	0.4	0.06	0.48	0.06	0.34^b^	0.09	0.44^b^	0.08	0.33^b^	0.08	0.67^a^	0.09	NS	∗	NS
Monocyte	0.13^c^	0.02	0.06^d^	0.01	0.10	0.02	0.08	0.02	0.11	0.02	0.1	0.02	∗∗∗	NS	NS
Basophil	0.03	0.01	0.02	0.01	0.04	0.01	0.02	0.01	0.03	0.01	0.02	0.01	NS	NS	∗∗
Plasma components															
Protein (g/L)	71.4	0.60	72.4	0.60	70.9^b^	0.87	70.8^b^	0.83	72.0^b^	0.82	74.1^a^	0.86	NS	∗	NS
Fibrinogen (g/L)	3.3	0.16	3.7	0.15	3.5	0.23	3.4	0.21	3.8	0.21	3.1	0.22	NS	NS	NS

Means with different superscripts are significantly different at *P* < 0.05, protected Fisher's LSD test.

^x^Significance *P < 0.05; **P < 0.01; ***P < 0.001.

**Table 2:** Haematological physiological means of sheep compared in the dry and wet seasons of Central Trinidad.

Blood	Growing		Dry pregnant		Lactating		Dry nonpregnant		Significance	
Parameters	Dry	Wet	Dry	Wet	Dry	Wet	Dry	Wet		
*N* Parameters	22	19	20	25	22	24	22	20	LSD	^ x^Significance

Red blood cells										
Haemoglobin (g/L)	98.4	103.7	91	89.6	97.7	94.4	89.1	87.2	9.06	NS
Packed cell volume (L/L)	0.30	0.31	0.27	0.27	0.29	0.28	0.27	0.26	0.027	NS
Mean corpuscular haemoglobin (g/L)	333	338	336	337	336	338	336	335	5.0	NS
Serum Cu (mg/L)	0.42	0.58	0.43	0.62	0.48	0.57	0.35	0.53	0.133	NS
White blood cells (×10^9^/L)										
White blood cell count	13.5	10.2	11.4	9.8	11.7	10.1	10.1	9.4	2.18	NS
Neutrophil	4.7	4.4	4.9	3.9	5.5	4.4	4.0	3.2	1.28	NS
Lymphocyte	7.9^a^	5.2^b^	5.5^b^	4.9^b^	4.9^b^	4.8^b^	4.9^b^	5.2^b^	1.41	∗
Eosinophil	0.33	0.34	0.43	0.46	0.33	0.32	0.53	0.82	0.33	NS
Monocyte	0.13	0.07	0.10	0.05	0.19	0.04	0.11	0.10	0.08	NS
Basophil	0.07^a^	0.01^b^	0.01^b^	0.03^b^	0.04^b^	0.02^b^	0.01^b^	0.25^b^	0.04	∗∗
Plasma components										
Protein (g/L)	70.1	71.7	69.8	71.8	72.8	71.2	72.9	75.3	3.36	NS
Fibrinogen (g/L)	3.3	3.7	3.3	3.6	3.6	4.1	3.0	3.2	0.87	NS

Means with different superscripts are significantly different at *P* < 0.05, protected Fishers LSD test.

^x^Significance *P < 0.05; **P < 0.01; NS: not significant.
